# Propofol Alleviates DNA Damage Induced by Oxygen Glucose Deprivation and Reperfusion *via* FoxO1 Nuclear Translocation in H9c2 Cells

**DOI:** 10.3389/fphys.2019.00223

**Published:** 2019-03-15

**Authors:** Dandan Zhou, Jinqiang Zhuang, Yihui Wang, Dandan Zhao, Lidong Zhao, Shun Zhu, Jinjun Pu, Ming Yin, Hongyu Zhang, Zejian Wang, Jiang Hong

**Affiliations:** ^1^ Department of Internal and Emergency Medicine, Shanghai General Hospital, Shanghai Jiao Tong University, Shanghai, China; ^2^ School of Pharmacy, Shanghai Jiao Tong University, Shanghai, China; ^3^ Department of Emergency Medicine, Putuo Hospital Affiliated to Shanghai University of Traditional Chinese Medicine, Shanghai, China; ^4^ Department of Biomedicine, KG Jebsen Centre for Research on Neuropsychiatric Disorders, University of Bergen, Bergen, Norway

**Keywords:** propofol, oxygen glucose deprivation and reperfusion, ROS, DNA damage, FoxO1

## Abstract

Ischemia/reperfusion (I/R) injury induces irreversible oxidative stress damage to the cardiac myocytes. Many studies have revealed that propofol alleviates the important organelle-mediated injury from oxidative stress *in vitro*. However, it remains unclear whether propofol prevents I/R-induced DNA damage in cardiomyocytes. In our study, we established an oxygen glucose deprivation/reoxygenation (OGD/R) model in H9c2 cells and found that propofol decreased reactive oxygen species (ROS) levels and suppressed cell apoptosis induced by OGD/R in H9c2 cells. In addition, propofol significantly reduced the molecular marker of DNA damage and inhibited double-strand breaks of DNA damage induced by OGD/R in H9c2 cells in a dose-dependent manner. Furthermore, we investigated the molecular mechanisms and demonstrated that propofol inhibited forkhead box O 1 (FoxO1) phosphorylation and increased FoxO1 nuclear translocation through inhibition of protein kinase B (Akt) and adenosine 5’-monophosphate-activated protein kinase (AMPK) pathways. The protective effects of propofol against oxidative stress-induced DNA damage were reversed by silencing FoxO1. Taken together, our results suggest that oxidative stress aggravates DNA damage and apoptosis in H9C2 cells, which can be reversed by propofol *via* FoxO1 nuclear translocation.

## Introduction

Myocardial ischemia injury followed by reperfusion induces irreversible oxidative stress damage and cardiomyocyte cell death (Murphy and Steenbergen, 2008). However, the underlying mechanism of ischemia/reperfusion (I/R) injury remains to be elucidated. A large amount of evidence suggests that pharmacological intervention for cardioprotection, which may offer novel therapeutic candidates to ameliorate the risk and progression of ischemic heart disease, and heart failure (Thibault et al., 2008; Hausenloy and Yellon, 2011). However, clinically available agents for the patient with ischemic heart disease are limited.

Propofol (2-6-diisopropylphenol) is commonly used in clinical anesthesia induction (4–6 μg/ml) and maintenance as well as in sedation (2–3 μg/ml) (Chidambaran et al., 2015). It has been shown that propofol exhibited cardioprotective effects in both cell and animal studies (Xia et al., 2003; Zhao et al., 2015). In addition, propofol inhibits I/R injuries in various experimental animal and cellular models by reducing the generation of ROS, scavenging free radicals, protecting the cell membrane and mitochondrial function from lipid peroxidation, and suppressing apoptosis (Corcoran et al., 2006; Jovic et al., 2012; Li et al., 2015). Prolonged production of ROS causes irreversible damage not only to cell membrane, organelles, but also to the nucleus. It remains unexplored whether propofol protects against DNA damage induced by ischemia/reperfusion in cardiomyocytes.

Myocardial I/R can produce a large number of ROS, which can cause damage to protein, lipid modifications, and DNA (Finkel and Holbrook, 2000). The aberrant DNA structures generated upon DNA damage can trigger DNA damage response (DDR), which refers to a network of cellular processes in order to detect and repair DNA lesions. The failure of DDR and gene repair can cause secondary myocardial cell apoptosis and even senescence, which leads to heart failure and even death (Bersell et al., 2013).

Three major antioxidant pathways (NF-E2-related factor-2 (Nrf2), Sirtuin 1 (SIRT1), and FoxO1) play an important role in the DDR (DeNicola et al., 2011; Pardo et al., 2011; Sengupta et al., 2011). We hypothesize that propofol may alleviate DNA damage induced by I/R injury *via* antioxidant pathways. In the present study, we used H9c2 cell line subjected to oxygen glucose deprivation and reperfusion (OGD/R) as an *in vitro* model of cardiomyocyte ischemia and investigated the potential mechanism underlying the cardioprotective effect of propofol against I/R.

## Materials and Methods

### Reagents and Antibodies

Dulbecco’s modified Eagle’s medium/F-12 (DMEM/F-12) and fetal bovine serum were purchased from Gibco-Invitrogen (Grand Island, NY, USA). Propofol and Dimethyl sulfoxide were purchased from Sigma-Aldrich (St. Louis, MO, USA). Akt inhibitor (wortmannin) and AMPK inhibitor (dorsomorphin) were purchased from Selleck. Akt activator (IGF-1) and AMPK activator (AICAR) were purchased from RD Systems. Antibodies against FoxO1 and Nrf2 were purchased from Abcam. Antibodies against BAX, phospho-ATM (Ser1981), phospho-ATR (Ser428), γ-H2AX (Ser139), phospho-CHK1 (Ser345), phospho-CHK2 (Thr68), phospho-P53 (Ser15), phospho-BRCA1 (Ser1524), Akt, phospho-Akt (Thr308), IRS-1, phospho-IRS-1 (Ser636/639), AMPK, phospho-AMPK (Thr172), and phospho-FoxO1 (Ser256) were purchased from Cell Signaling Technology (Danvers, MA, USA). Antibodies against NOX2, SIRT1, Cat, SOD1, Histone-H3, and GAPDH were purchased from Proteintech (Chicago, IL, USA).

### Cell Culture

Rat cardiomyocyte H9c2 cell line was purchased from Shanghai Institute for Biological Sciences, Chinese Academy of Science (Shanghai, China). The cells were cultured in DMEM/F-12 supplemented with 10% fetal bovine serum and 1% penicillin/streptomycin at 37 °C in a humidified incubator containing 5% CO_2_.

### Oxygen Glucose Deprivation/Reoxygenation (OGD/R) Model and Drug Treatment

Oxygen glucose deprivation/reoxygenation model and drug treatment were performed as previously described (Zhao et al., 2015). Briefly, cells were exposed to hypoxic conditions (oxygen deprivation, 0.5% O_2_) for 24 h in culture medium deprived of glucose and combined with 1% fetal bovine serum. After hypoxia, the cells were oxygenated under normoxic conditions (reoxygenation) for 24 h in normal medium. Propofol with different concentrations (5, 10, 20, and 40 μM) was added, respectively, to the cells 1 h before and during the hypoxia-reoxygenation.

### Cell Viability Assay

Cell viability was measured by the methylthiazolyldiphenyl-tetrazolium bromide (MTT; Beyotime, Haimen, China) method. Cells were seeded in a 96-well cell at a density of 2 × 10^4^ cells/well. After 24 h of culture, cells were treated with propofol or dimethyl sulfoxide for hypoxia-oxygenation, respectively. Then, 10 μl of MTT solution was added to each well at the final concentration of 0.5 mg/ml and incubated for 4 h at 37 °C. A 100 ml dimethyl sulfoxide was then added to dissolve formazan crystals, and the absorbance at 570 nm was measured using an AMR-100 automatic enzyme analyzer (Allsheng, Hangzhou, China).

### Intracellular ROS Detection

Cells were seeded in a 96-well plate at a density of 3 × 10^4^ cells/well. After 24 h of incubation, the cells were exposed to OGD condition for 24 h and subsequently treated with propofol at 20 μM concentration under reoxygenation condition for 12 h. For the detection of intracellular ROS, the cells were preloaded with 10 μM of 2,7-dichlorofluorescin diacetate (DCFH-DA, Beyotime, Haimen, China) for 20 min at 37 °C, and then, the plates were washed using DMEM without serum five times at least. A fluorescence microplate reader with an excitation wavelength of 488 nm and an emission wavelength of 525 nm was used to determine the intensity of DCF fluorescence.

### Cell Apoptosis Assay

Cells were seeded into a 6-well plate and treated as described in oxygen glucose deprivation/reoxygenation model and drug treatment above. Annexin V-FITC Apoptosis Detection Kit (Beyotime, Haimen, China) was used for the detection of apoptotic cells according to the manufacturer’s protocol. The proportion of apoptotic cells was calculated by FlowJo software.

### Cytoplasmic and Nuclear Protein Extraction

This assay was conducted by using NE-PER Nuclear and Cytoplasmic Extraction Reagents Kit (Thermo Scientific, USA) according to the manufacturers’ protocol. Briefly, the supernatant was carefully removed, and the cell pellet was left as dry as possible. CER I was added to the cell pellet, incubating for 10 min. Then, CER II was added, and supernatant (cytoplasmic extract) was collected after vortex and centrifugation. NER was added to the cell pellet, and nuclear extract was collected in the same way. The volume ratio of CER I:CER II:NER reagents was at 200:11:100, and all the procedures were performed on ice with the reagent being pre-cold.

### FoxO1-Specific siRNA Silenced FoxO1

H9c2 cells were seeded in a 6-well plate at 5 × 10^6^ cells/well and incubated at 37 °C and 5% CO_2_. According to the manufacturer’s instructions, three different specific siRNA oligonucleotides (50 nM) or the scrambler oligonucleotides as control (provided by the Shanghai Tuo Ran biological company) were transfected into H9c2 cells with Lipofectamine 2000 to knockout FoxO1 in the following day. Six hours after transfection, the cells were updated with normal medium. The transfection reagent used in this study was the levels of FoxO1 protein in different clones that were determined by the western blot analysis. The FoxO1 knockdown siRNA:

Rn-FoxO1-si-1: 5′-CCAGGCACCUCAUAACAAA-3′
Rn-FoxO1-si-2: 5′-CAUGACAGCAAAGUGCCAA-3′
Rn-FoxO1-si-3: 5′-CAAGUCUUGUAUAUAUGCA-3′

### Western Blotting

Cells were harvested and washed with cold phosphate buffered saline (PBS). Cells were lysed with RIPA buffer containing protease and phosphatase inhibitor cocktails (Roche, Germany). Insoluble material was removed by centrifugation at 16,000 rpm for 20 min at 4 °C. The supernatants were collected and quantified for protein concentration with bicinchoninic acid (BCA) kit (Beyotime, Haimen, China) according to the manufacturer’s instructions. Total proteins from cell lysates were denatured at 100°C for 5 min; separated on 6, 10, and 12% SDS-PAGE; and transferred to polyvinylidene difluoride membranes (PVDF, Millipore, Billerica, MA, USA). The membranes were blocked with 5% BSA (albumin from bovine serum) in TBS containing 0.1% Tween-20 (TBST) for 2 h at room temperature and then incubated sequentially with primary antibodies at 4 °C overnight. The primary antibodies included goat GAPDH and Histone-H3, γ-H2AX, p-ATM, p-ATR, p-CHK1, p-CHK2, p-P53, p-BRCA1, BAX, NOX2, SIRT1, Nrf2, FoxO1, p-FoxO1, Akt, p-Akt, IRS-1, p-IRS-1, AMPK, p-AMPK, SOD1, and Cat. After primary antibody incubation, the membranes were washed with TBST three times and incubated with either goat anti-mouse or goat anti-rabbit horseradish peroxidase-conjugated secondary antibodies at a dilution of 1:5,000 for 2 h at room temperature. Washed with TBST five times, the membranes were developed with electrochemiluminescence (ECL) reagent (Thermo-Pierce, Rockford, IL, USA). The density of immunoblotting bands was quantified using Gel-Pro Analyzer software (Media Cybernetics, Silver Spring, MD, USA).

### Statistical Analysis

All experiments were repeated at least three times. The results were presented as the mean ± standard deviation (SD). Statistical analysis was performed using the software Statistical Package for the Social Science (SPSS, Chicago, IL, USA). Quantitative data were analyzed by one-way analysis of variance (ANOVA). Student-Newman-Keuls test was used for *post hoc* analysis to identify significant differences between groups. Differences with *p* < 0.05 were considered statistically significant.

## Results

### Propofol Decreased ROS Levels and Inhibited Cell Apoptosis Induced by OGD/R in H9c2 Cells

**Figure 1:**
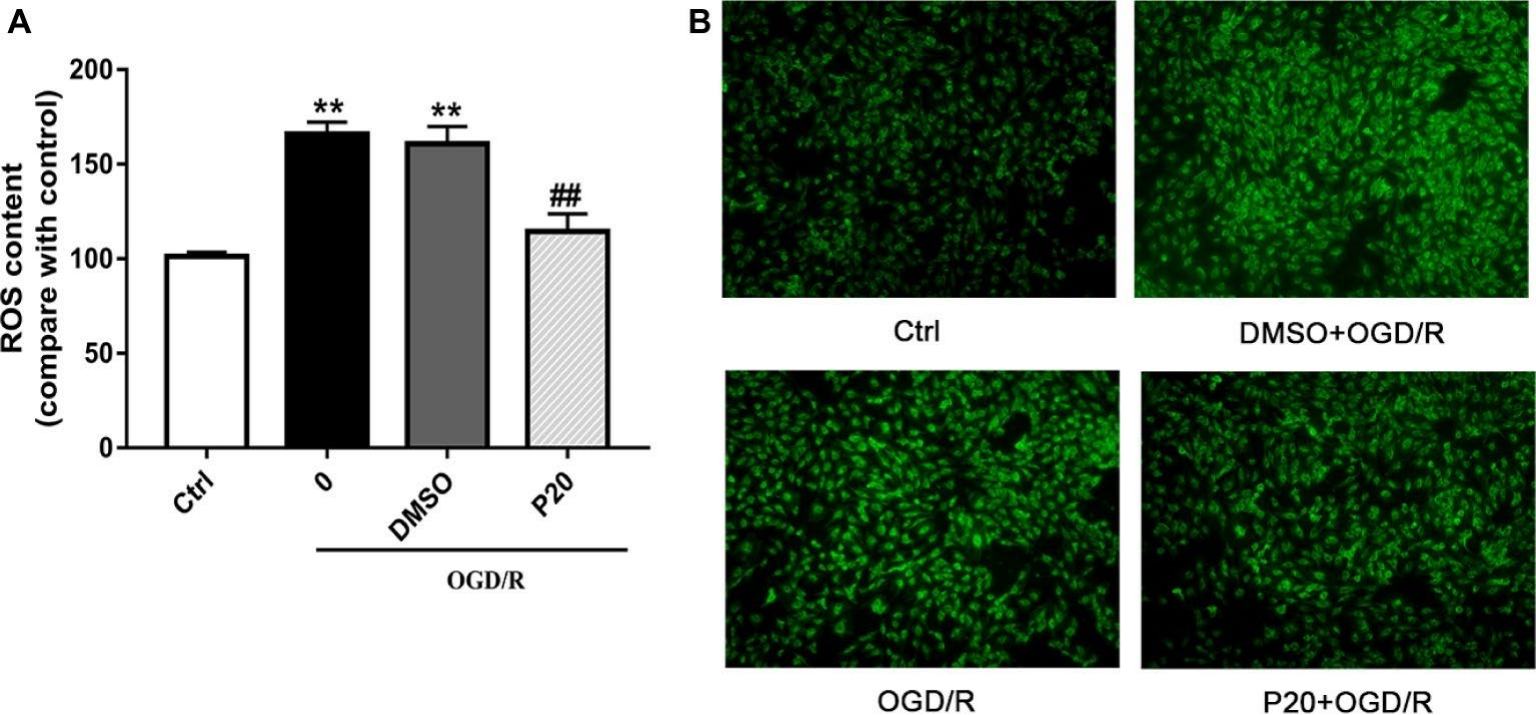
Effects of propofol on ROS contents during OGD/R in H9c2 cells. DMSO and propofol adopted during the entire ischemia/reperfusion phase. The ctrl group was defined as 100%. **(A)** Effects of propofol on OGD/R induced intracellular ROS contents increase in H9c2 cells. **(B)** Representative DCF fluorescent images, ×100. The results were shown as mean ± SD from three independent experiments. ^*^ *p* < 0.05, ^**^ *p* < 0.01, ^***^ *p* < 0.001 versus control, ^#^ *p* < 0.05, ^##^ *p* < 0.01, ^###^ *p* < 0.001 versus OGD/R treated group without drugs.

**Figure 2:**
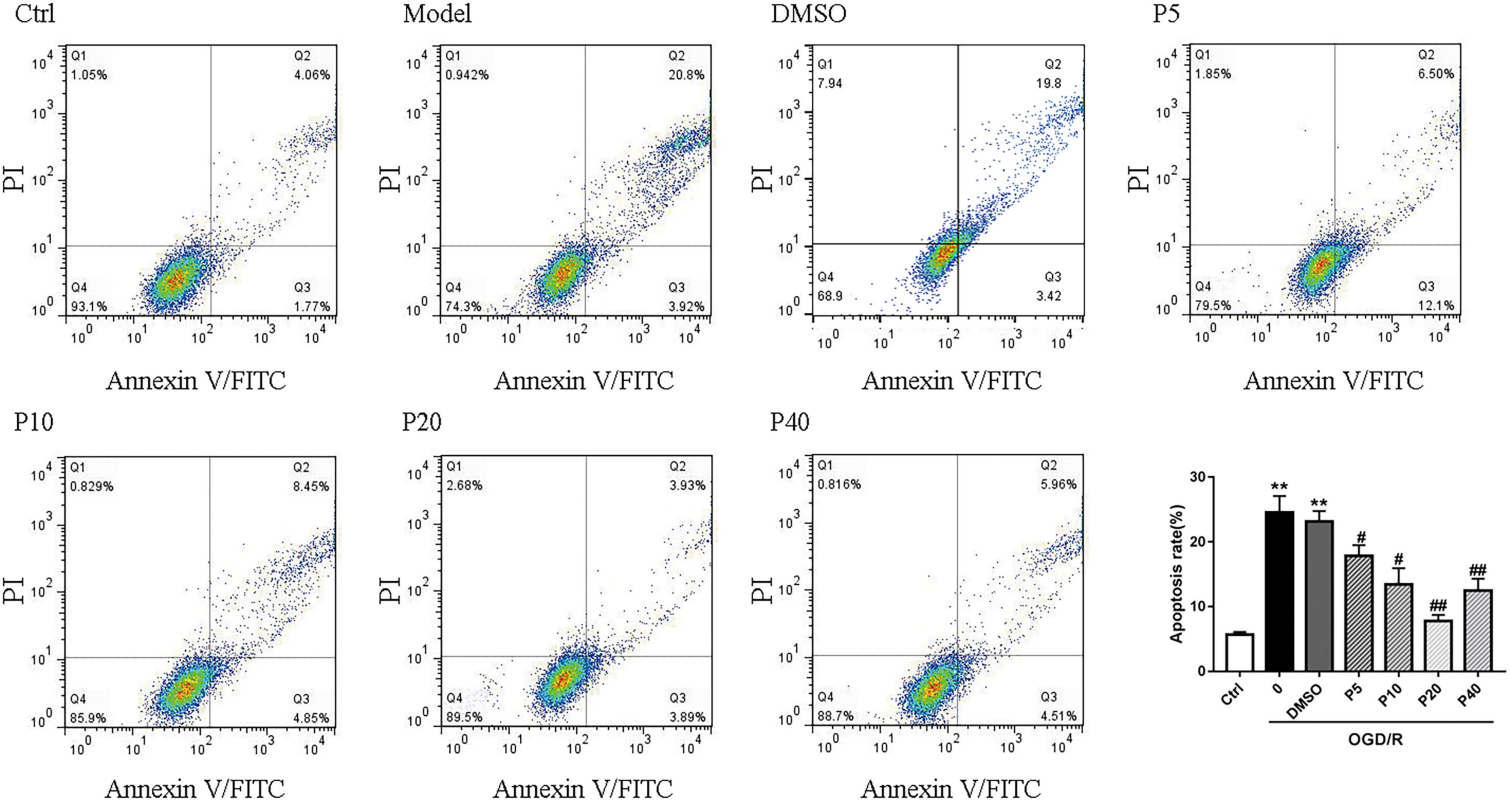
Propofol inhibited cell apoptosis induced by OGD/R in H9c2 cells. Quantification of the apoptotic cell population by flow cytometry. Propofol decreased the percentage of apoptotic cells compared with the model. The data are presented as the mean ± SD of three independent experiments. ^*^ *p* < 0.05, ^**^ *p* < 0.01, ^***^ *p* < 0.001 versus control, ^#^ *p* < 0.05, ^##^ *p* < 0.01, ^###^ *p* < 0.001 versus OGD/R treated group without drugs.

As depicted in Figures 1 and 2, OGD/R significantly elevated intracellular ROS level and cell apoptosis compared with control. Administration of propofol at 20 μM concentration profoundly prevented the OGD/R-induced increase in ROS level (Figures 1A,B). Consistently, the apoptosis rates were also decreased after the application of propofol (Figure 2). These results demonstrated that propofol can prevent the OGD/R-induced increase in the intracellular ROS level and cell apoptosis rate in H9c2 cells.

### Propofol Inhibited DNA Double-Strand Breaks Induced by OGD/R in H9c2 Cells

**Figure 3:**
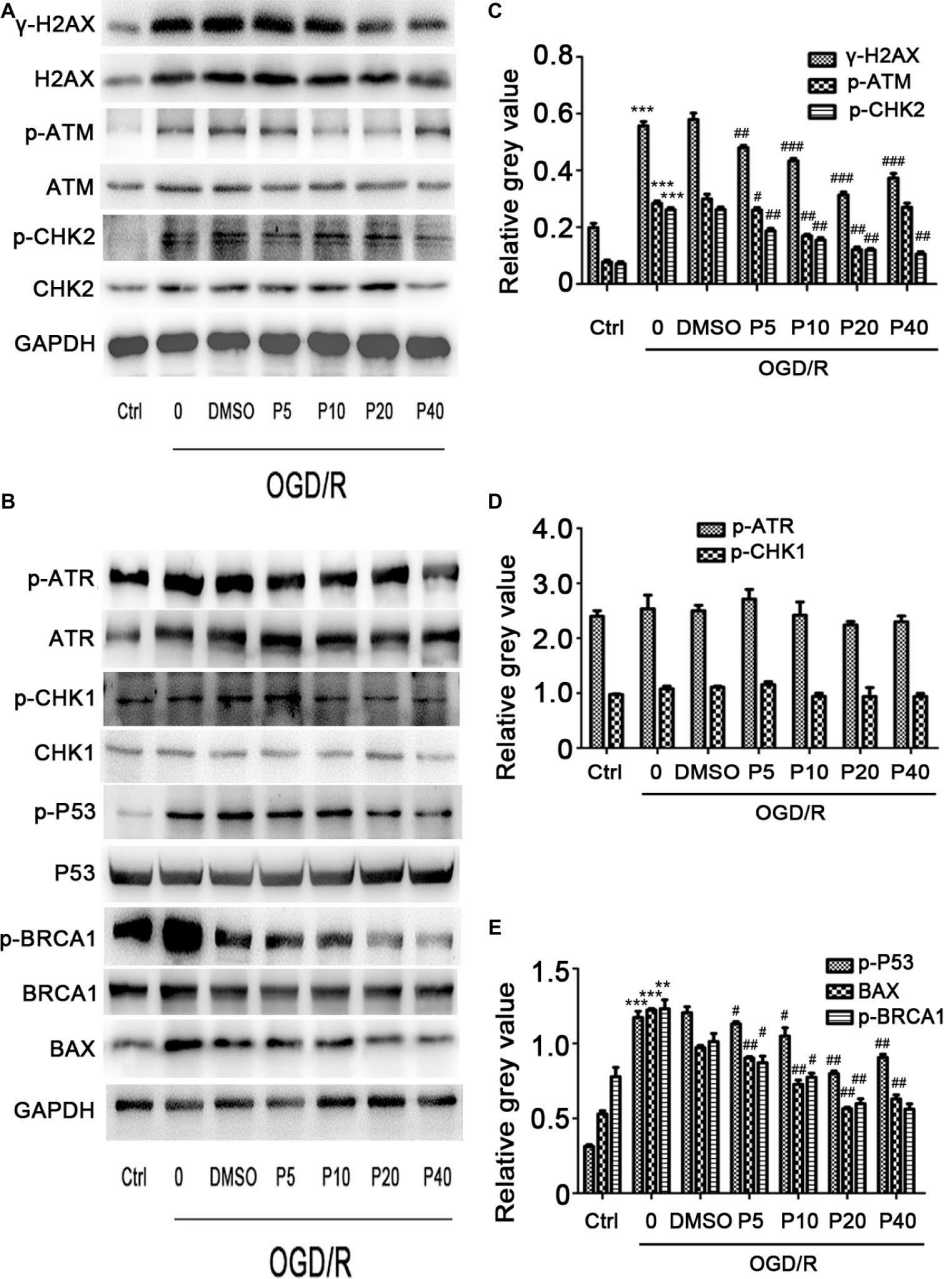
The effects of propofol on the expression of DNA double-strand break-related proteins induced by OGD/R in H9c2 cells during OGD/IR insult. Representative bands of **(A)** γ-H2AX, p-ATM, p-CHK2, **(B)** p-ATR, p-CHK1, p-BRCA1, p-P53, Bax by western blotting. **(C–E)** Relative gray value of γ-H2AX, p-ATM, p-CHK2, p-ATR, p-CHK1, p-BRCA1, p-P53, and Bax. GAPDH was used as an internal control. The data are presented as the mean ± SD of three independent experiments. ^*^ *p* < 0.05, ^**^ *p* < 0.01, ^***^ *p* < 0.001 versus control, ^#^ *p* < 0.05, ^##^ *p* < 0.01, ^###^ *p* < 0.001 versus OGD/R treated group without drugs.

We next examined whether propofol could ameliorate the DNA damage induced by oxidative stress in OGD/R. As shown in Figures 3A,C, the phosphorylation level of H2AX, ATM, and CHK2 was markedly elevated after OGD/R exposure, which was significantly reversed by the application of propofol (5, 10, 20, and 40 μM). However, phosphorylation level of ATR and CHK1 was comparable between the groups (Figures 3B,D).

### Effect of Propofol on OGD/R-Induced DNA Damage in H9c2 Cells

We evaluated p-P53 and BAX expression using the western blotting. Compared with the control group, the relative gray value of p-P53, BAX was significantly increased during OGD/R insult in model group, and the level of p-P53 and BAX in propofol groups was decreased in a dose-dependent manner (Figures 3B,E). We also measured the p-BRCA1 activation by using the relative gray value/total in each group. Compared with model group, propofol treatment greatly decreased the expression of p-BRCA1 (Figure 3B).

### Effect of Propofol on OGD/R-Induced Cell Death Through the Regulation of FoxO1 to Reduce ROS in H9c2 Cells

**Figure 4:**
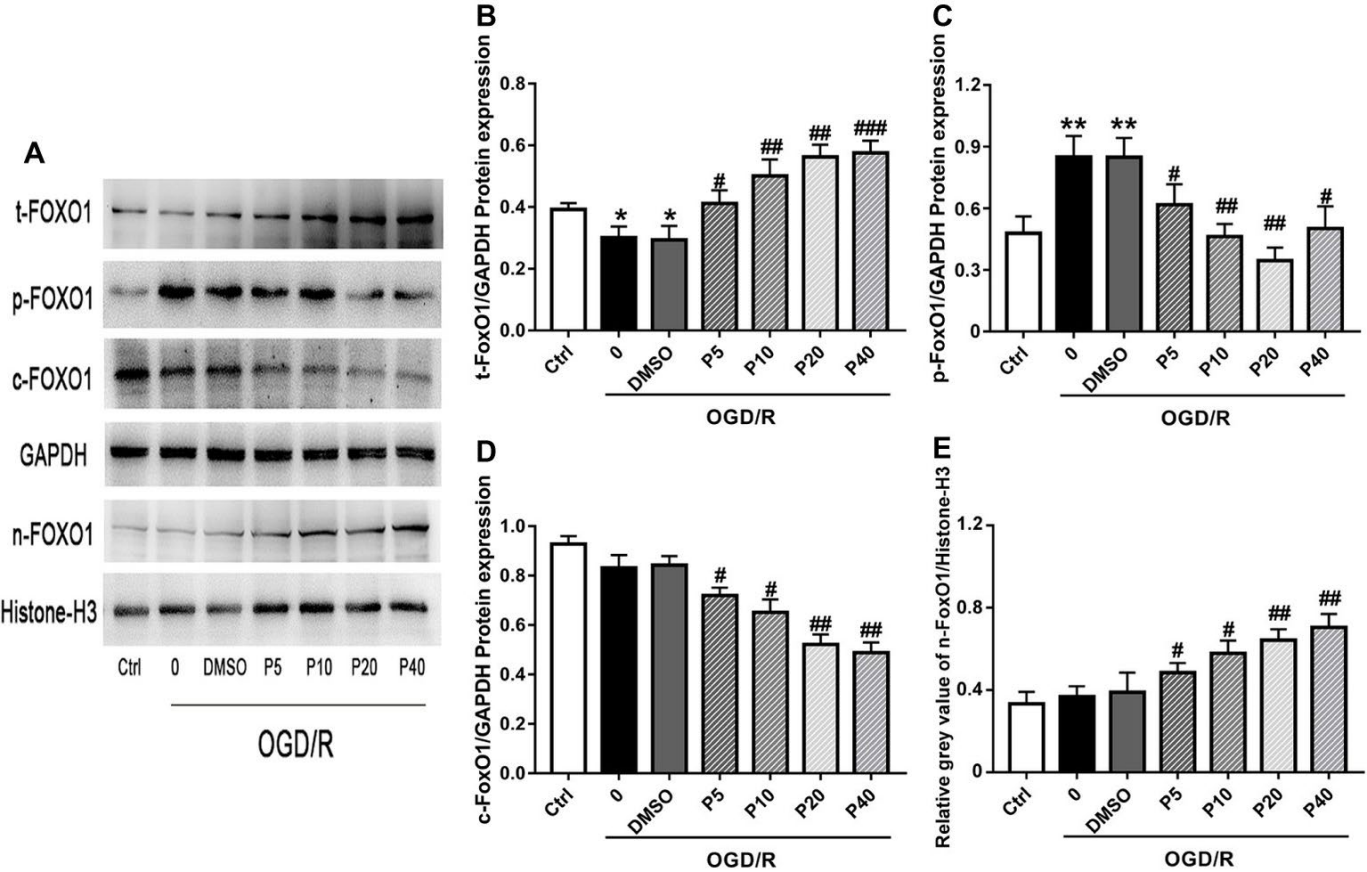
Propofol inhibited FoxO1 phosphorylation and promoted FoxO1 shuttling into the nucleus in H9c2 cells during OGD/R insult. Representative bands of **(A)** t-FoxO1, p-FoxO1, c-FoxO1, and n-FoxO1. Relative gray value/GAPDH of **(B)** t-FoxO1, **(C)** p-FoxO1, **(D)** c-FoxO1, **(E)** n-FoxO1. The data are presented as the mean ± SD of three independent experiments. ^*^ *p* < 0.05, ^**^ *p* < 0.01, ^***^ *p* < 0.001 versus control, ^#^ *p* < 0.05, ^##^ *p* < 0.01, ^###^ *p* < 0.001 versus OGD/R treated group without drugs.

NOX2 is considered one of the major producers of ROS, which play an important role in intracellular ROS homeostasis (Bedard and Krause, 2007). Nrf2, SIRT1, and FoxO1 play an important role in cellular adaptation to oxidative stress through regulation of antioxidant proteins, such as SOD1 and Cat, to reduce ROS (Greer and Brunet, 2005; DeNicola et al., 2011; Pardo et al., 2011). There was no significant statistical difference of the expression of NOX2, Nrf2, or SIRT1, among the groups (Data not shown). Compared with the model group, the levels of total-FoxO1 and nucleus-FoxO1 in propofol groups were increased in a dose-dependent manner (Figures 4A,B,E). However, the levels of cytoplasm-FoxO1 and phospho-FoxO1 in propofol groups were decreased (Figures 4A,C,D).

### FoxO1 Silencing Aggravates the OGD/R-Induced DNA Damage in H9c2 Cell

**Figure 5:**
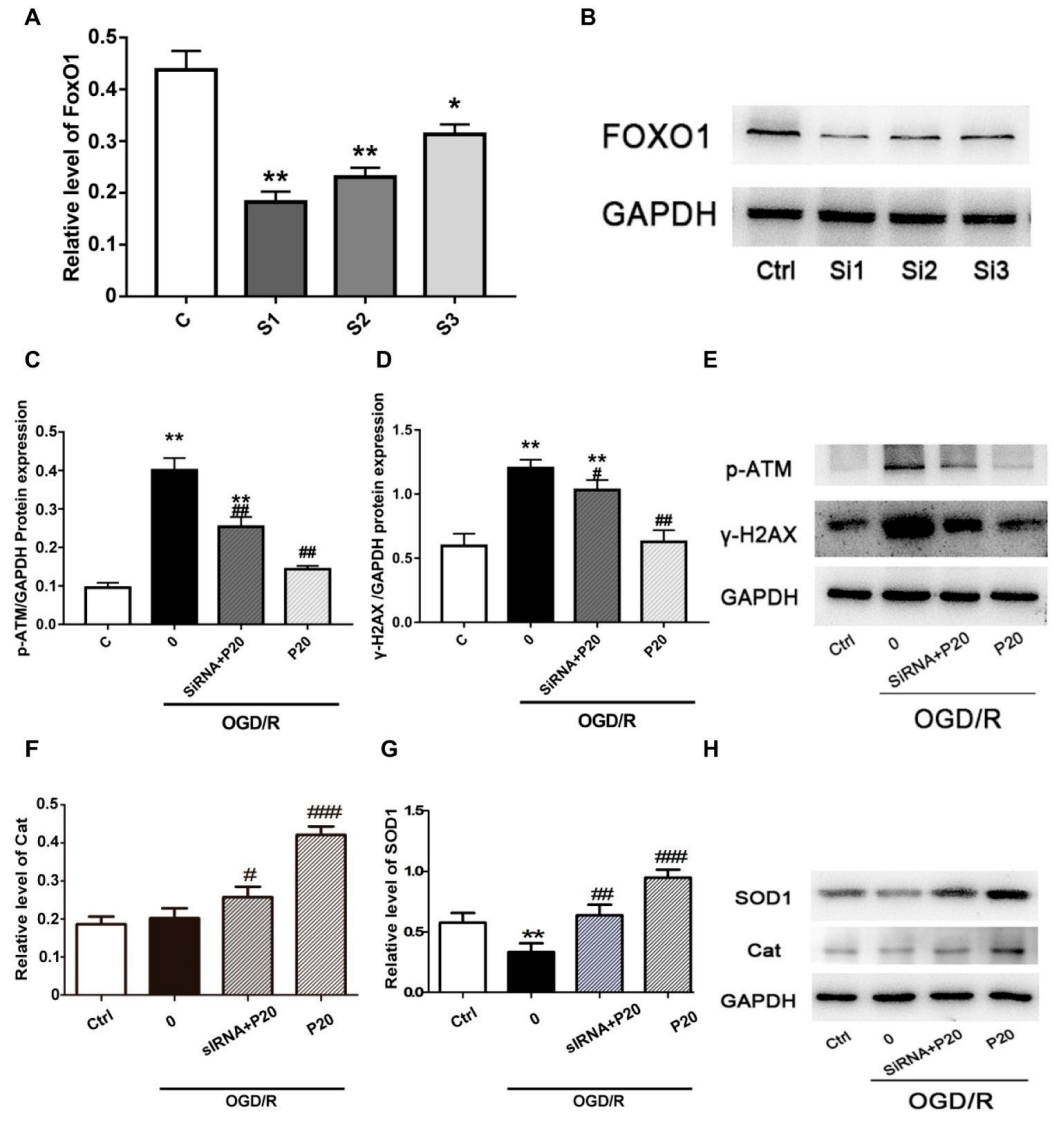
The FoxO1 silence significantly reversed the effect of propofol on DSBs in H9c2 cells during OGD/R insult. **(A)** and **(B)** Representative image of FoxO1 silence vesicles in H9c2 cells, which was, respectively, transfected with 50 nM siRNA-FoxO1–1, siRNA-FoxO1–2, or siRNA-FoxO1–3. **(C)**, **(D)**, **(F)**, and **(G)**: Relative level of γ-H2AX, p-ATM, Cat, and SOD1 in the OGD/R induced and siRNA-transfected H9c2 cells. **(E)** and **(H)** Expression of γ-H2AX, p-ATM, Cat, and SOD1 in protein level in H9c2 cells treated with 20 μM propofol, 50 nM siRNA-FoxO1–1, or both of them. The data are presented as the mean ± SD of three independent experiments. ^*^ *p* < 0.05, ^**^ *p* < 0.01, ^***^ *p* < 0.001 versus control, ^#^ *p* < 0.05, ^##^ *p* < 0.01, ^###^ *p* < 0.001 versus OGD/R treated group without drugs.

To identify the role of FoxO1 in mediating the oxidative stress damage or anti-oxidative stress progress in H9c2 cell, we silenced FoxO1 using FoxO1-specific siRNA in H9c2 cells and evaluated the effects of propofol on OGD/R-induced DNA damage. We synthesized three sequences of siRNA-FoxO1 and screened out that transfection with 50 nM siRNA-FoxO1–1 markedly downregulated the level of FoxO1 expression (Figures 5A,B). Interestingly, after the significant knockdown of FoxO1 with FoxO1-specific siRNA, the levels of γ-H2AX and p-ATM expression in the OGD/R-induced and siRNA-FoxO1-transfected H9c2 cells were increased (Figures 5C–E), whereas the levels of Cat and SOD1 expression were decreased (Figures 5F–H).

**Figure 6:**
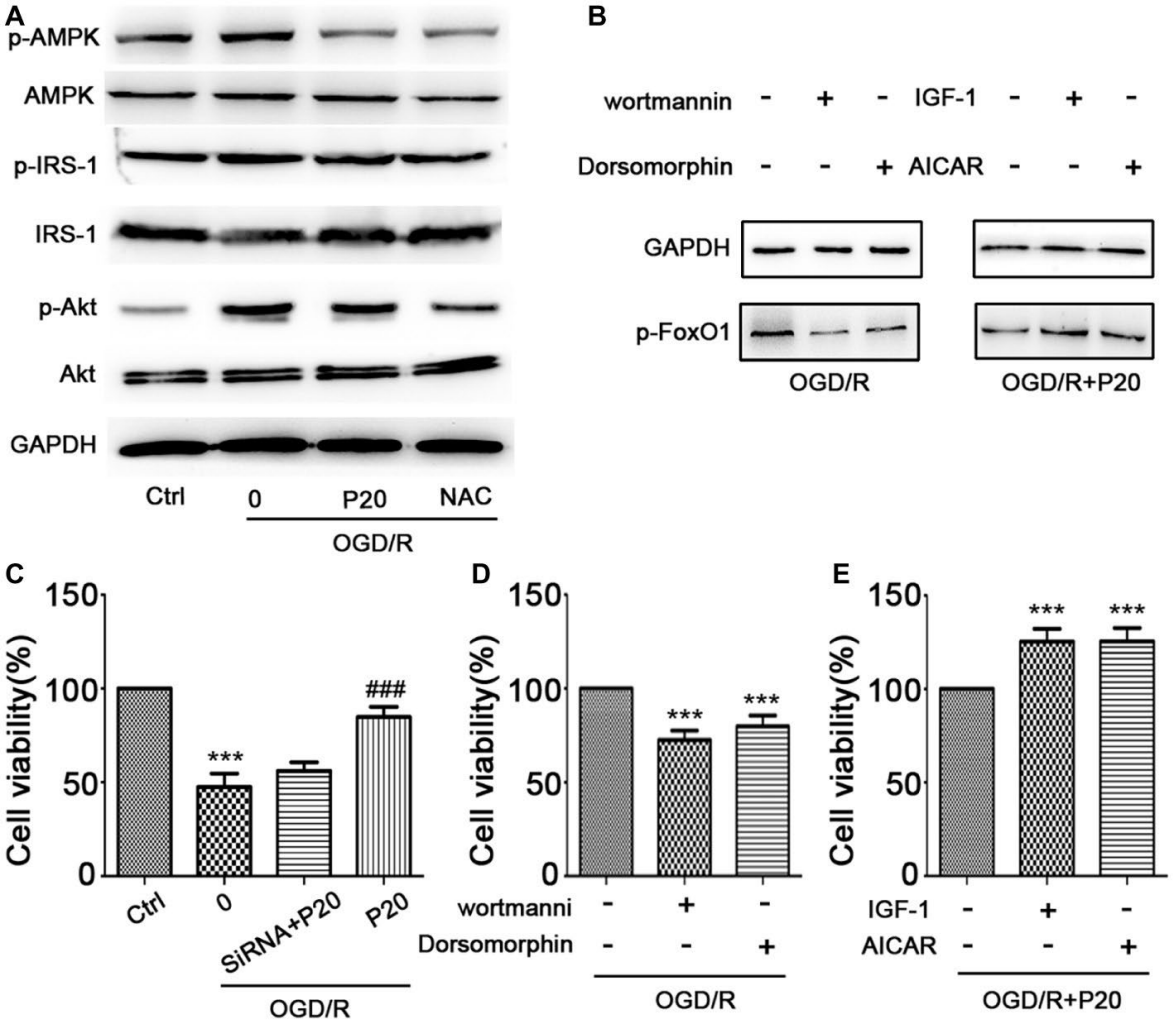
Propofol inhibited FoxO1 phosphorylation through Akt and AMPK pathways. **(A)** The expression of proteins in FoxO1-related pathways in H9c2 cells. **(B)** The expression of p-FoxO1 after being treated with inhibitors and activators of Akt and AMPK pathways. **(C)** Cell viability was assessed by MTT assay after FoxO1 siRNA transfection in H9c2 cells. **(D–E)** Cell viability was assessed by MTT assay after being treated with inhibitors and activators of Akt and AMPK pathways. The data are presented as the mean ± SD of three independent experiments. ^*^ *p* < 0.05, ^**^ *p* < 0.01, ^***^ *p* < 0.001 versus control, ^#^ *p* < 0.05, ^##^ *p* < 0.01, ^###^ *p* < 0.001 versus OGD/R treated group without drugs.

**Figure 7:**
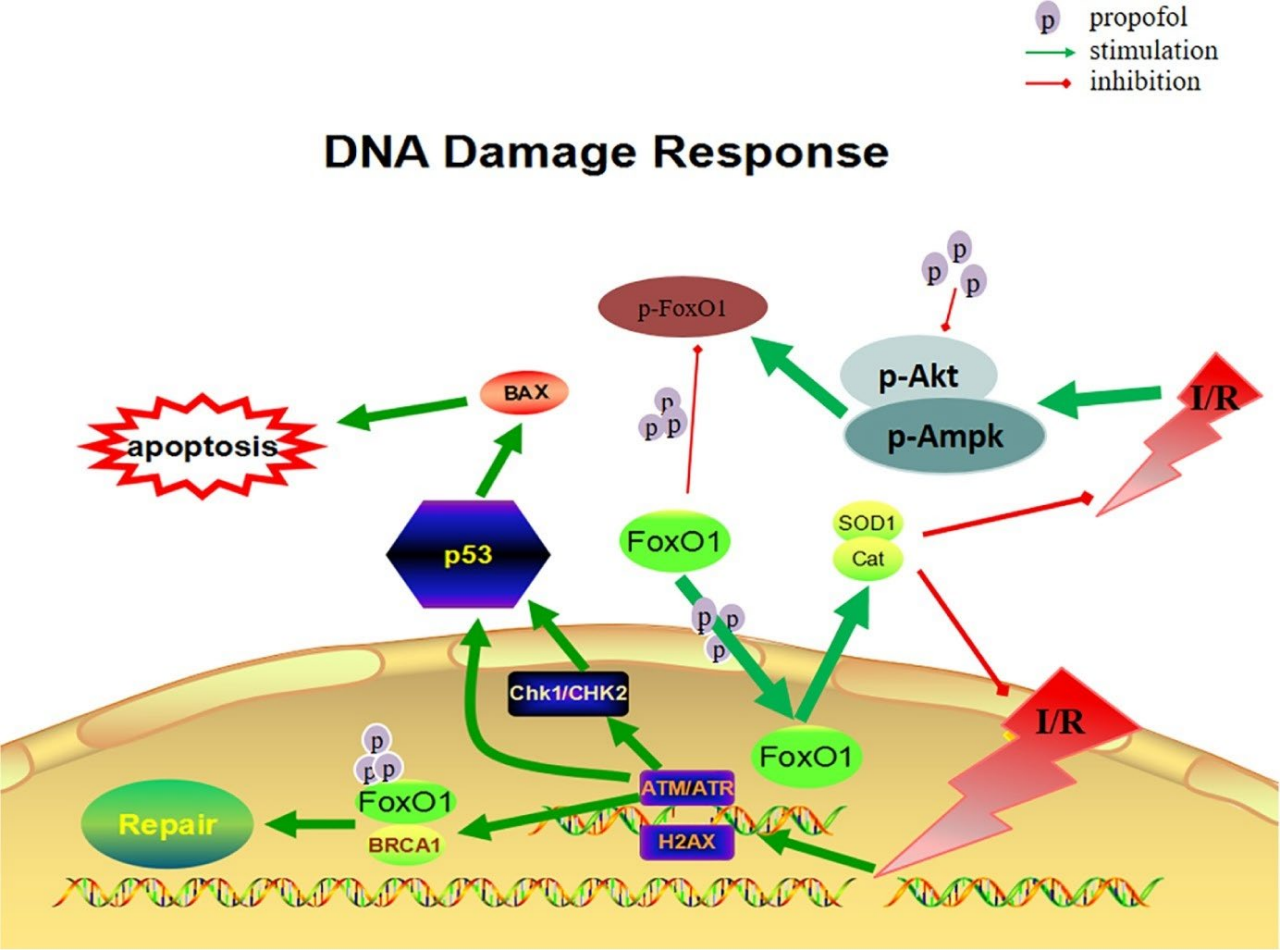
Diagram of DNA damage response *via* FoxO1 pathways.

### Protective Effects of Propofol Against OGD/R-Induced DNA Damage *via* Inhibition of AMPK and Akt Pathway

The modulation of FoxO1 involves in Akt (Zhang et al., 2018) and AMPK (Liu et al., 2018) pathways. We thus evaluated the expression of the proteins in these pathways. We demonstrated that OGD/R exposure elevated the phosphorylation level of Akt and AMPK, which was reversed by treatment with propofol in H9c2 cells (Figure 6A). We further used inhibitors (wortmannin and dorsomorphin) and activators (IGF-1 and AICAR) of Akt and AMPK pathway to treat the cells and found that both inhibitors decreased the expression of p-FoxO1, while activators increased the expression of p-FoxO1 (Figure 6B), indicating that the inhibition of both Akt and AMPK pathways mediates the protective effects of propofol against OGD/R-induced DNA damage, which is consistent with the changes in cell viability detected by MTT assay (Figures 6C–E).

## Discussion

Our study elucidated a mechanism underlying the protective effect of propofol against OGD/R-induced DNA damage, possibly *via* FoxO1 nuclear translocation in H9c2 cells. We first established an *in vitro* model of myocardial cell injury induced by OGD/R in H9c2 cells. We demonstrated that propofol enhanced the survival of H9c2 cells in a dose-dependent manner and decreased ROS level during OGD/R insult.

ROS are critically involved in the I/R injury (Finkel and Holbrook, 2000; Eltzschig and Eckle, 2011). Increased ROS can cause DNA damage (Finkel and Holbrook, 2000). DDR involves complex signaling pathways (Sancar et al., 2004), which can be coordinated primarily by two distinct kinase signaling cascades, the ataxia telangiectasia mutated/checkpoint kinase 2 (ATM–CHK2) and the Rad3-related protein/checkpoint kinase 1 (ATR–CHK1) pathways, activated by DNA double-strand breaks (DSBs) and single-stranded DNA, respectively (Smith et al., 2010). γ-H2AX, a phosphorylated histone variant H2AX at serine139, is widely believed to be a sensor of DNA damage signaling. γ-H2AX plays an important role in recruiting the DDR proteins to the DNA lesion sites and initiating the DDR, including DNA repair and cell cycle checkpoints (Paull et al., 2000; Rappold et al., 2001; Huen et al., 2010). We found that the OGD/R-induced increase in phosphorylation of ATM, CHK2, and H2AX was reversed by propofol in a dose-dependent manner, while the level of phosphorylation of ATR and CHK1 remained unchanged. We also showed that OGD/R caused upregulation in the expression of p53 and p-BRCA1 in H9c2 cells was prevented by propofol dose-dependently in comparison with the control group. These results suggested that ROS induced by OGD/R insult mainly caused DNA double-strand breaks, which led to upregulated expression of p53 and cell apoptosis. In addition, DNA repair was activated through ATM-CHK2 pathway. Propofol significantly inhibited the production of ROS and promoted DNA repair in H9c2 cells, protecting cardiomyocyte from ischemic injury.

The production of ROS is related to many factors, and the increase of NADPH oxidase is one of the major sources (Hahn et al., 2011). NOX2, also known as gp91phox, is the prototype NADPH oxidase. NOX2 antisense strongly inhibited hypoxia-induced oxidative stress and apoptosis in cardiomyocytes *in vitro*. Propofol failed to inhibit the expression of NOX2 after OGD/R insult, suggesting that propofol might not directly inhibit the production of ROS.

Sirtuin 1 (SIRT1) and NF-E2-related factor-2 (Nrf2) are critical regulators of cytoprotective proteins during oxidative stress (Evans-Anderson et al., 2008; DeNicola et al., 2011; Pardo et al., 2011). However, we did not detect the activation of SIRT1 and Nrf2 signaling pathways in cardiomyocytes during the OGD/R insult. FoxOs belong to the forkhead family of transcriptional regulators, including FoxO1, FoxO3, FoxO4, and FoxO6 (Evans-Anderson et al., 2008) of which FoxO1 is highly expressed in adult cardiomyocytes. The FoxO1 plays an important role in cellular adaptation to oxidative stress through regulation of antioxidant genes as well as transactivating ROS-detoxifying enzymes (Furukawa-Hibi et al., 2002; van den Berg and Burgering, 2011). The phosphorylation of FoxO1 prevents its translocation to the nucleus and blocks its function. In our study, we found that propofol enhanced the protein expression of t-FoxO1 and n-FoxO1 and decreased the expression of c-FoxO1 and p-FoxO1 induced by OGD/R. In addition, the FoxO1 silence significantly reversed the protective effect of propofol against DSBs induced by OGD/R in H9c2 cells. Therefore, propofol inhibited FoxO1 phosphorylation and promoted FoxO1 shuttle into the nucleus, which may help increase expression of antioxidants, decrease production of ROS, and alleviate DNA damage and cell death.

Forkhead box O regulation can be principally achieved by post-translational modifications or protein-protein interactions (Daitoku et al., 2011). FoxO1 has been reported to be modulated by Nrf2 and SIRT1 signaling pathways (Lee and Goldberg, 2013; Gille et al., 2019). FoxO1 is involved in various pathways, including Akt (Zhang et al., 2018) and AMPK (Liu et al., 2018) pathways. We demonstrated that propofol reversed the elevated phosphorylation level of Akt and AMPK pathways induced by OGD/R in H9c2 cells. Inhibitors (wortmannin and dorsomorphin) of Akt and AMPK decreased the expression of p-FoxO1, while the activators (IGF-1 and AICAR) increased the expression of p-FoxO1, indicating that both Akt and AMPK pathways were involved in protection of propofol against DNA damage induced by OGD/R, which was consistent with the changes in cell viability detected by MTT assay. It has been reported that propofol reduced oxidative stress through FoxO1 and AMPK pathways (Zhao et al., 2015). But, it was controversial that only 10 μM of propofol decreased the p-AMPK level. A possible explanation is that there are more ways than one by which FoxO1 is phosphorylated. However, in our study, we actually found that there was no significant crosstalk between FoxO1 and Nrf2 (or SIRT1) in the protection of propofol against DNA damage induced by OGD/R. It has been reported that both Nrf2 and SIRT1 can be activated by AMPK signaling pathway. Since propofol significantly decreased the level of Akt and AMPK phosphorylation during oxidative stress, it is possible that propofol may mainly promote nuclear translocation of the FoxO1 *via* Akt and AMPK signaling pathways, instead of Nrf2 or SIRT1. GSK3β is also a cardioprotective enzyme. It is previously reported that GSK3β plays an important role in propofol cardioprotection. In our study, there is no evidence that GSK3β mediates the protective effect of propofol against DNA damage (data not shown).

## Conclusions

The cardioprotective effect of propofol against OGD/R injury in the H9c2 was through the inhibition of FoxO1 phosphorylation and the promotion of FoxO1 shuttle into the nucleus *via* Akt and AMPK pathways, which helps to alleviate DNA damage and cell death by sequentially increasing the expression of antioxidants enzymes and decreasing the production of ROS correspondingly (Figure 7).

## Data Availability

All datasets generated for this study are included in the manuscript and/or the supplementary files.

## Author Contributions

JH and ZW designed the research. DZho, JZ, YW, and DZha performed the research. MY contributed new reagents or analytic tools. LZ, SZ, and JP analyzed the data. DZho wrote the manuscript. HZ contributed to revising it critically for important intellectual content. All authors have read and approved of the version to be published.

### Conflict of Interest Statement

The authors declare that the research was conducted in the absence of any commercial or financial relationships that could be construed as a potential conflict of interest.

## References

[ref1] Bedard K. Krause K. H. (2007). The NOX family of ROS-generating NADPH oxidases: physiology and pathophysiology. Physiol. Rev. 87, 245–313. doi: 10.1152/physrev.00044.2005, PMID: 17237347

[ref2] Bersell K. Choudhury S. Mollova M. Polizzotti B. D. Ganapathy B. Walsh S.. (2013). Moderate and high amounts of tamoxifen in alphaMHC-MerCreMer mice induce a DNA damage response, leading to heart failure and death. Dis. Model. Mech. 6, 1459–1469. doi: 10.1242/dmm.010447, PMID: 23929941 PMC3820268

[ref3] Chidambaran V. Costandi A. D’mello A. (2015). Propofol: a review of its role in pediatric anesthesia and sedation. CNS Drugs 29, 543–563. doi: 10.1007/s40263-015-0259-6, PMID: 26290263 PMC4554966

[ref4] Corcoran T. B. Engel A. Sakamoto H. O’shea A. O’callaghan-Enright S. Shorten G. D. (2006). The effects of propofol on neutrophil function, lipid peroxidation and inflammatory response during elective coronary artery bypass grafting in patients with impaired ventricular function. Br. J. Anaesth. 97, 825–831. doi: 10.1093/bja/ael270, PMID: 17032661

[ref5] Daitoku H. Sakamaki J. Fukamizu A. (2011). Regulation of FoxO transcription factors by acetylation and protein-protein interactions. Biochim. Biophys. Acta 1813, 1954–1960. doi: 10.1016/j.bbamcr.2011.03.001 21396404

[ref6] DeNicola G. M. Karreth F. A. Humpton T. J. Gopinathan A. Wei C. Frese K.. (2011). Oncogene-induced Nrf2 transcription promotes ROS detoxification and tumorigenesis. Nature 475, 106–109. doi: 10.1038/nature10189, PMID: 21734707 PMC3404470

[ref7] Eltzschig H. K. Eckle T. (2011). Ischemia and reperfusion—from mechanism to translation. Nat. Med. 17, 1391–1401. doi: 10.1038/nm.2507, PMID: 22064429 PMC3886192

[ref8] Evans-Anderson H. J. Alfieri C. M. Yutzey K. E. (2008). Regulation of cardiomyocyte proliferation and myocardial growth during development by FOXO transcription factors. Circ. Res. 102, 686–694. doi: 10.1161/CIRCRESAHA.107.163428, PMID: 18218983

[ref9] Finkel T. Holbrook N. J. (2000). Oxidants, oxidative stress and the biology of ageing. Nature 408, 239–247. doi: 10.1038/35041687, PMID: 11089981

[ref10] Furukawa-Hibi Y. Yoshida-Araki K. Ohta T. Ikeda K. Motoyama N. (2002). FOXO forkhead transcription factors induce G(2)-M checkpoint in response to oxidative stress. J. Biol. Chem. 277, 26729–26732. doi: 10.1074/jbc.C200256200, PMID: 12048180

[ref11] Gille A. Turkistani A. Tsitsipatis D. Hou X. Tauber S. Hamann I.. (2019). Nuclear trapping of inactive FOXO1 by the Nrf2 activator diethyl maleate. Redox Biol. 20, 19–27. doi: 10.1016/j.redox.2018.09.010, PMID: 30261343 PMC6156746

[ref12] Greer E. L. Brunet A. (2005). FOXO transcription factors at the interface between longevity and tumor suppression. Oncogene 24, 7410–7425. doi: 10.1038/sj.onc.1209086, PMID: 16288288

[ref13] Hahn N. E. Meischl C. Wijnker P. J. Musters R. J. Fornerod M. Janssen H. W.. (2011). NOX2, p22phox and p47phox are targeted to the nuclear pore complex in ischemic cardiomyocytes colocalizing with local reactive oxygen species. Cell. Physiol. Biochem. 27, 471–478. doi: 10.1159/000329968, PMID: 21691064

[ref14] Hausenloy D. J. Yellon D. M. (2011). The therapeutic potential of ischemic conditioning: an update. Nat. Rev. Cardiol. 8, 619–629. doi: 10.1038/nrcardio.2011.85, PMID: 21691310

[ref15] Huen M. S. Sy S. M. Chen J. (2010). BRCA1 and its toolbox for the maintenance of genome integrity. Nat. Rev. Mol. Cell Biol. 11, 138–148. doi: 10.1038/nrm2831, PMID: 20029420 PMC3899800

[ref16] Jovic M. Stancic A. Nenadic D. Cekic O. Nezic D. Milojevic P.. (2012). Mitochondrial molecular basis of sevoflurane and propofol cardioprotection in patients undergoing aortic valve replacement with cardiopulmonary bypass. Cell. Physiol. Biochem. 29, 131–142. doi: 10.1159/000337594, PMID: 22415082

[ref17] Lee D. Goldberg A. L. (2013). SIRT1 protein, by blocking the activities of transcription factors FoxO1 and FoxO3, inhibits muscle atrophy and promotes muscle growth. J. Biol. Chem. 288, 30515–30526. doi: 10.1074/jbc.M113.489716, PMID: 24003218 PMC3798522

[ref18] Li Y. Zhong D. Lei L. Jia Y. Zhou H. Yang B. (2015). Propofol prevents renal ischemia-reperfusion injury via inhibiting the oxidative stress pathways. Cell. Physiol. Biochem. 37, 14–26. doi: 10.1159/000430329, PMID: 26277932

[ref19] Liu J. Q. Zhang L. Yao J. Yao S. Yuan T. (2018). AMPK alleviates endoplasmic reticulum stress by inducing the ER-chaperone ORP150 via FOXO1 to protect human bronchial cells from apoptosis. Biochem. Biophys. Res. Commun. 497, 564–570. doi: 10.1016/j.bbrc.2018.02.095, PMID: 29448096

[ref20] Murphy E. Steenbergen C. (2008). Mechanisms underlying acute protection from cardiac ischemia-reperfusion injury. Physiol. Rev. 88, 581–609. doi: 10.1152/physrev.00024.2007, PMID: 18391174 PMC3199571

[ref21] Pardo P. S. Mohamed J. S. Lopez M. A. Boriek A. M. (2011). Induction of Sirt1 by mechanical stretch of skeletal muscle through the early response factor EGR1 triggers an antioxidative response. J. Biol. Chem. 286, 2559–2566. doi: 10.1074/jbc.M110.149153, PMID: 20971845 PMC3024751

[ref22] Paull T. T. Rogakou E. P. Yamazaki V. Kirchgessner C. U. Gellert M. Bonner W. M. (2000). A critical role for histone H2AX in recruitment of repair factors to nuclear foci after DNA damage. Curr. Biol. 10, 886–895. doi: 10.1016/S0960-9822(00)00610-2, PMID: 10959836

[ref23] Rappold I. Iwabuchi K. Date T. Chen J. (2001). Tumor suppressor p53 binding protein 1 (53BP1) is involved in DNA damage-signaling pathways. J. Cell Biol. 153, 613–620. doi: 10.1083/jcb.153.3.613, PMID: 11331310 PMC2190566

[ref24] Sancar A. Lindsey-Boltz L. A. Unsal-Kacmaz K. Linn S. (2004). Molecular mechanisms of mammalian DNA repair and the DNA damage checkpoints. Annu. Rev. Biochem. 73, 39–85. doi: 10.1146/annurev.biochem.73.011303.073723, PMID: 15189136

[ref25] Sengupta A. Molkentin J. D. Paik J. H. Depinho R. A. Yutzey K. E. (2011). FoxO transcription factors promote cardiomyocyte survival upon induction of oxidative stress. J. Biol. Chem. 286, 7468–7478. doi: 10.1074/jbc.M110.179242, PMID: 21159781 PMC3045002

[ref26] Smith J. Tho L. M. Xu N. Gillespie D. A. (2010). The ATM-Chk2 and ATR-Chk1 pathways in DNA damage signaling and cancer. Adv. Cancer Res. 108, 73–112. doi: 10.1016/B978-0-12-380888-2 21034966

[ref27] Thibault H. Piot C. Staat P. Bontemps L. Sportouch C. Rioufol G.. (2008). Long-term benefit of postconditioning. Circulation 117, 1037–1044. doi: 10.1161/CIRCULATIONAHA.107.729780, PMID: 18268150

[ref28] van den Berg M. C. Burgering B. M. (2011). Integrating opposing signals toward Forkhead box O. Antioxid. Redox Signal. 14, 607–621. doi: 10.1089/ars.2010.3415, PMID: 20624032

[ref29] Xia Z. Godin D. V. Ansley D. M. (2003). Propofol enhances ischemic tolerance of middle-aged rat hearts: effects on 15-F(2t)-isoprostane formation and tissue antioxidant capacity. Cardiovasc. Res. 59, 113–121. doi: 10.1016/S0008-6363(03)00351-1, PMID: 12829182

[ref30] Zhang S. Chen X. Huang Z. Chen D. Yu B. He J.. (2018). Effects of microrna-27a on myogenin expression and Akt/FoxO1 signal pathway during porcine myoblast differentiation. Anim. Biotechnol. 29, 183–189. doi: 10.1080/10495398.2017.1348357 28799830

[ref31] Zhao D. Li Q. Huang Q. Li X. Yin M. Wang Z.. (2015). Cardioprotective effect of propofol against oxygen glucose deprivation and reperfusion injury in H9c2 cells. Oxidative Med. Cell. Longev. 2015:184938. doi: 10.1155/2015/184938, PMID: . 25821553 PMC4364303

