# Corrigendum: Propofol Alleviates DNA Damage Induced by Oxygen Glucose Deprivation and Reperfusion *via* FoxO1 Nuclear Translocation in H9c2 Cells

**DOI:** 10.3389/fphys.2020.00059

**Published:** 2020-02-06

**Authors:** Dandan Zhou, Jinqiang Zhuang, Yihui Wang, Dandan Zhao, Lidong Zhao, Shun Zhu, Jinjun Pu, Ming Yin, Hongyu Zhang, Zejian Wang, Jiang Hong

**Affiliations:** ^1^Department of Internal and Emergency Medicine, Shanghai General Hospital, Shanghai Jiao Tong University, Shanghai, China; ^2^School of Pharmacy, Shanghai Jiao Tong University, Shanghai, China; ^3^Department of Emergency Medicine, Putuo Hospital Affiliated to Shanghai University of Traditional Chinese Medicine, Shanghai, China; ^4^Department of Biomedicine, KG Jebsen Centre for Research on Neuropsychiatric Disorders, University of Bergen, Bergen, Norway

**Keywords:** propofol, oxygen glucose deprivation and reperfusion, ROS, DNA damage, FoxO1

In the original article, there was a mistake in Figure 6 as published. In Part A, the images of IRS-1 and p-IRS-1 were the same as the images of AMPK and p-AMPK. The corrected Figure 6 appears below.

**Figure 6:**
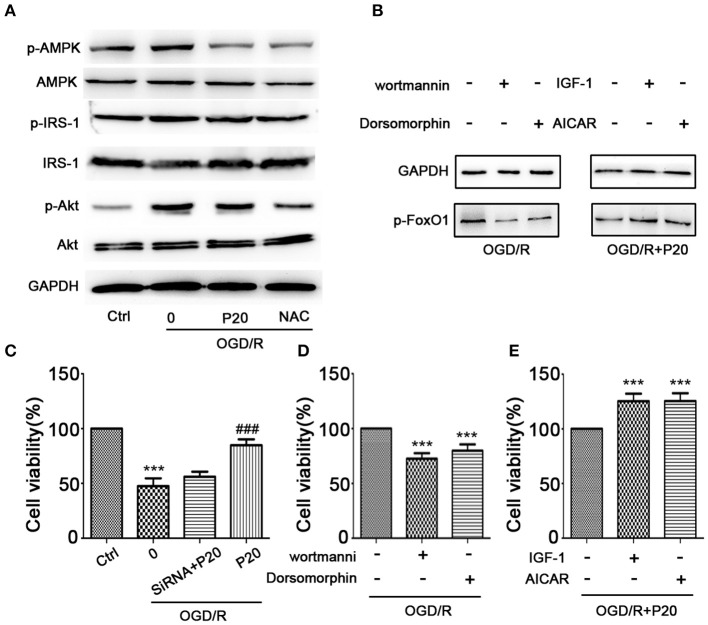
Propofol inhibited FoxO1 phosphorylation through Akt and AMPK pathways. **(A)** The expression of proteins in FoxO1-related pathways in H9c2 cells. **(B)** The expression of p-FoxO1 after being treated with inhibitors and activators of Akt and AMPK pathways. **(C)** Cell viability was assessed by MTT assay after FoxO1 siRNA transfection in H9c2 cells. **(D–E)** Cell viability was assessed by MTT assay after being treated with inhibitors and activators of Akt and AMPK pathways. The data are presented as the mean ± SD of three independent experiments. *p < 0.05, **p < 0.01, ***p < 0.001 versus control, ^#^*p* < 0.05, ^*##*^*p* < 0.01, ^*###*^*p* < 0.001 versus OGD/R treated group without drugs.

The authors apologize for this error and state that this does not change the scientific conclusions of the article in any way. The original article has been updated.

