# Corrigendum: Propofol Alleviates DNA Damage Induced by Oxygen Glucose Deprivation and Reperfusion *via* FoxO1 Nuclear Translocation in H9c2 Cells

**DOI:** 10.3389/fphys.2022.805972

**Published:** 2022-03-29

**Authors:** Dandan Zhou, Jinqiang Zhuang, Yihui Wang, Dandan Zhao, Lidong Zhao, Shun Zhu, Jinjun Pu, Ming Yin, Hongyu Zhang, Zejian Wang, Jiang Hong

**Affiliations:** ^1^Department of Internal and Emergency Medicine, Shanghai General Hospital, Shanghai Jiao Tong University, Shanghai, China; ^2^School of Pharmacy, Shanghai Jiao Tong University, Shanghai, China; ^3^Department of Emergency Medicine, Putuo Hospital Affiliated to Shanghai University of Traditional Chinese Medicine, Shanghai, China; ^4^Department of Biomedicine, KG Jebsen Centre for Research on Neuropsychiatric Disorders, University of Bergen, Bergen, Norway

**Keywords:** propofol, oxygen glucose deprivation and reperfusion, ROS, DNA damage, FoxO1

In Figure 2 of the article, we provided the wrong image of the DMSO group. The correct Figure 2 is displayed below.

**Figure 2:**
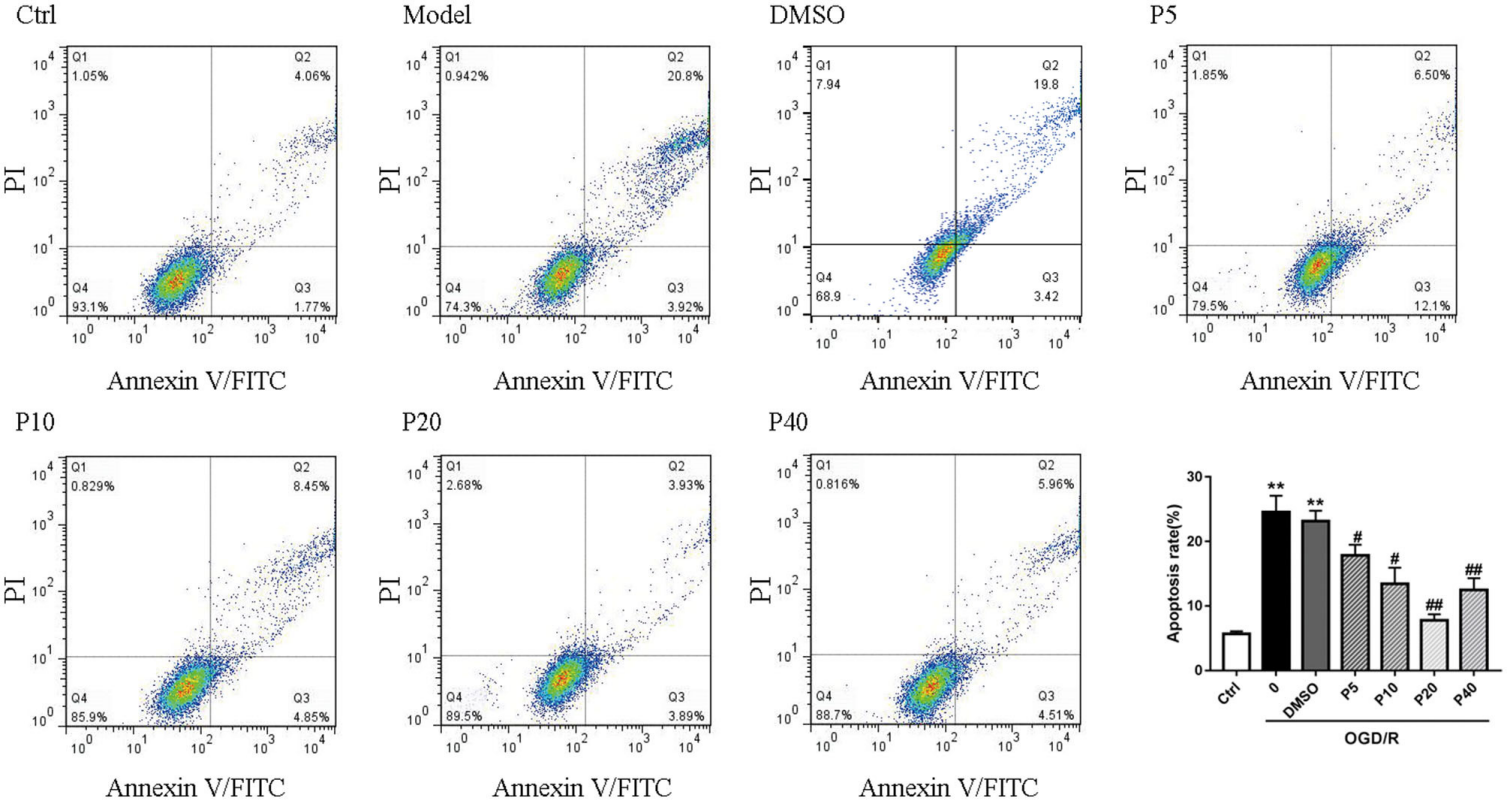
Propofol inhibited cell apoptosis induced by OGD/R in H9c2 cells. Quantification of the apoptotic cell population by flow cytometry. Propofol decreased the percentage of apoptotic cells compared with the model. The data are presented as the mean ± SD of three independent experiments. *p < 0.05, **p < 0.01, ***p < 0.001 versus control, ^#^*p* < 0.05, ^##^*p* < 0.01, ^###^*p* < 0.001 versus OGD/R treated group without drugs.

The authors apologize for this error and state that this does not change the scientific conclusions of the article in any way. The original article has been updated.

## Publisher's Note

All claims expressed in this article are solely those of the authors and do not necessarily represent those of their affiliated organizations, or those of the publisher, the editors and the reviewers. Any product that may be evaluated in this article, or claim that may be made by its manufacturer, is not guaranteed or endorsed by the publisher.

